# Effects of Acoustic Modulation and Mixed Fuel on Flame Synthesis of Carbon Nanomaterials in an Atmospheric Environment

**DOI:** 10.3390/ma9110939

**Published:** 2016-11-18

**Authors:** Wei-Chieh Hu, Shanti Kartika Sari, Shuhn-Shyurng Hou, Ta-Hui Lin

**Affiliations:** 1 Department of Mechanical Engineering, National Cheng Kung University, Tainan 70101, Taiwan; n18981018@mail.ncku.edu.tw (W.-C.H.); shantikartikasari@gmail.com (S.K.S.); 2 Department of Mechanical Engineering, Kun Shan University, Tainan 71070, Taiwan; 3 Research Center for Energy Technology and Strategy, National Cheng Kung University, Tainan 70101, Taiwan

**Keywords:** flame synthesis, carbon nanotubes, carbon nano-onions, acoustic excitation, mixed fuel

## Abstract

In this study, methane–ethylene jet diffusion flames modulated by acoustic excitation in an atmospheric environment were used to investigate the effects of acoustic excitation frequency and mixed fuel on nanomaterial formation. Acoustic output power was maintained at a constant value of 10 W, while the acoustic excitation frequency was varied (*f* = 0–90 Hz). The results show that the flame could not be stabilized on the port when the ethylene volume concentration (Ω_E_) was less than 40% at *f* = 10 Hz, or when Ω_E_ = 0% (i.e., pure methane) at *f* = 90 Hz. The reason for this is that the flame had a low intensity and was extinguished by the entrained air due to acoustic modulation. Without acoustic excitation (*f* = 0 Hz), the flame was comprised of a single-layer structure for all values of Ω_E_, and almost no carbon nanomaterials were synthesized. However, with acoustic excitation, a double-layer flame structure was generated for frequencies close to both the natural flickering frequency and the acoustically resonant frequency. This double-layer flame structure provided a favorable flame environment for the fabrication of carbon nanomaterials. Consequently, the synthesis of carbon nano-onions was significantly enhanced by acoustic excitation near both the natural flickering frequency and the acoustically resonant frequency. At *f* = 20 Hz (near the natural flickering frequency) for 0% ≤ Ω_E_ ≤ 100%, a quantity of carbon nano-onions (CNOs) piled like bunches of grapes was obtained as a result of improved mixing of the fuel with ambient air. High-density CNOs were also produced at *f* = 70 Hz (close to the acoustically resonant frequency) for 40% ≤ Ω_E_ ≤ 100%. Furthermore, carbon nanotubes (CNTs) were synthesized only at 80 Hz for Ω_E_ = 0%. The suitable temperature range for the synthesis of CNTs was slightly higher than that for the formation of CNOs (about 600 °C for CNTs; 510–600 °C for CNOs).

## 1. Introduction

In the past few decades, great progress has been made in nanotechnology. The discovery and synthesis of novel carbon nanomaterials (CNMs) are two of the milestones. C_60_: Buckminsterfullerenes [1], carbon nanotubes (CNTs) [2], carbon nano-onions (CNOs) [3], graphene [4], and other CNMs have drawn much attention due to their unique mechanical, electrical, and chemical properties [5,6]. CNMs have been used for many electronic, optical and magnetic applications, such as gas sensors, high-temperature superconductors [7], scanning microscope tips, hydrogen storage media [8], and so on. The literature contains many proposals for CNT synthesis, including laser ablation [1], chemical vapor deposition (CVD) [2] and arc discharge [9]. However, these methods are expensive and complex. It has been shown that the flame synthesis of CNTs makes possible low cost, mass production of CNTs [8]. Accordingly, various flame synthesis methods for CNT production have been proposed in recent years.

Hydrocarbon flames can naturally and easily produce an appropriate high-temperature environment with the high radical concentrations required for the initiation and growth of carbon nanomaterials (CNMs) [10]. A metal catalyst introduced in the flame environment provides the reaction sites for the fabrication of CNMs, such as carbon nanotubes (CNTs), carbon nano-onions (CNOs), graphenes, carbon nanofibers, etc. Therefore, flame synthesis shows a more promising potential for inexpensive, rapid, mass production of CNMs than other synthesis methods. In recent years, great effort has been devoted to studying the synthesis of CNMs in flames [10,11,12,13].

Vander Wal et al. [14] synthesized single-walled CNTs in co-flow diffusion flames of ethylene/air and acetylene/air. They found that the yield of CNTs using acetylene was ten times more than using ethylene, and no CNTs were found in methane or nitrogen-diluted methane diffusion flames. Xu et al. [15] examined the effects of the addition of acetylene and different catalytic metal-alloys on CNT synthesis. A non-sooty flame (50% methane, 50% nitrogen) and a sooty flame (42% methane, 3% acetylene, 55% nitrogen) were employed while air was the oxidizer. They found that the addition of acetylene affected the morphology of CNTs for Ni/Cr/Fe and Ni/Ti alloys by aligning the CNTs.

Camacho and Choudhuri [16] investigated the effects of gaseous fuel type on the formation mechanism. In their study, CNTs as well as carbon nano-rods and carbon nano fibers could all be produced by using methane, but only CNTs were obtained when using propane. By altering the fuel to acetylene, helically coiled and twisted CNTs were produced.

Merchan-Merchan et al. [17,18,19] synthesized CNTs and other micro/nano-structures in a counterflow diffusion flame which consisted of 96% methane and 4% acetylene as the fuel and more than 50% oxygen diluted with nitrogen as the oxidizer. It was reported that an oxygen-rich flame has a strong potential for nanotube growth due to its high temperature and high radical concentrations [17]. CNTs at high oxygen concentrations (up to 68%) were synthesized without the use of a catalyst. Subsequently, they employed a metal probe as both the sampling substrate and catalyst [18]. The probe was an Ni-based alloy with 73% Ni, 17% Cu, and 10% Fe. The sampling time was 10 min. In a diffusion flame of 96% methane, 4% acetylene versus 50% oxygen and 50% nitrogen, the production rate and length of the CNTs were greatly improved compared to the results of their previous study. Furthermore, with an additional electric field, it was found that an external electric field could improve the alignment of CNTs in flame synthesis [19].

Xu et al. [20] synthesized CNTs using methane inverse diffusion flame. Three different catalytic probes were employed: 99.5% Fe; 45% Ni, 55% Cu; and 60% Ni, 16% Cr, 24% Fe. Samples were collected from different heights and radial positions referring to the burner exit. Their results showed that Fe was less conducive to the growth of CNTs and that the production and structure varied with the sampling position.

Yuan et al. [21] used a methane jet diffusion flame to fabricate CNTs. They found that the growth of CNTs was dependent on residence time, oxygen concentration and temperature. Samples were collected using an Ni-Cr wire (60% Ni, 26% Cr, 14% Fe), and the sampling time was 15–30 min. Abundant catalytic particles were observed for sampling times more than 10 min, but the formation of CNTs only took less than 1 min, which suggested that only a low concentration of the catalyst was required. The oxygen concentration contributed to the formation of catalytic particles from the substrate. However, oxidation of CNTs might occur under either high oxygen concentration or high temperature. They further investigated the effects of temperature by nitrogen dilution [22] using the experimental method similar to that in [21]. It was found that the diameter of the CNTs increased with increases in the temperature and that the sampling time affected the CNT yield. Similar results were also reported in ethylene diffusion flames [23].

Li et al. synthesized CNTs using methane flames in a counterflow configuration [24]. They examined the effects of substrate, temperature and strain rate on CNTs. Well-aligned CNTs were synthesized using 1D nano-templates, while entangled CNTs were found on Ni-alloy wires. A temperature window for CNTs was suggested, which is in a common range used in the CVD method (1023–1073 K). Moreover, they found that the strain rate (the residence time of the carbon sources) had almost no effect on CNT growth.

Du et al. [25] reported a one-step synthesis of CNTs grafted onto carbon fibers (CFs) with Ni catalysts in an ethanol flame. It was found that a high density of CNTs with diameters 5–20 nm and lengths up to 1 μm is uniformly grown on the surface of the CFs. Meanwhile, higher concentrations of the catalysts lead to thicker and denser growth of CNTs. In a recent study [26], CNTs were also grown in situ on CNFs at low temperature in an ethanol flame to develop multifunctional hierarchical reinforcements for epoxy resin matrices. No evident decrease of the tensile strength of the CFs was found due to the low temperature (about 450 °C), short duration and reducing atmosphere utilized in the flame synthesis. Moreover, both the electrical conductivity and interfacial properties of the CFs were markedly improved after the growth of CNTs for only 3 min.

The synthesis of CNOs in flames is very similar to that for CNTs. Factors such as temperature, fuel–oxygen ratio and gas composition dominate the growth of CNOs [27]. However, studies on CNOs fabricated by flame synthesis are relatively scarce. Silvestrini et al. [28] investigated the effects of acetylene and oxygen concentrations on CNO formation. The flames were methane–oxygen counterflow diffusion flames with the addition of 0%–4% acetylene in methane and varied oxygen concentrations of 21%–100% diluted with nitrogen. The highest yield was observed when soot formed substantially, which corresponded to flames with more acetylene. Liu and Li [29] synthesized CNOs and CNTs in acetylene-oxygen premixed flames. CNOs were synthesized without a catalyst, while CNTs were formed on a stainless steel mesh coated with CoCl_2_. An oxygen-enriched flame was found to lead to complete combustion and generated only water and carbon dioxide, while excess acetylene produced a large amount of amorphous carbon. A proper range of the acetylene-oxygen ratio is therefore required for CNOs.

Chung et al. [30] investigated the influence of acoustic modulation on the growth of CNOs in ethylene–air jet diffusion flames using an Ni substrate. At the axial position of *z* = 10 mm, the yield of CNOs occurred at high frequencies near the natural flickering frequency (10–30 Hz), at which the gas temperature was in the range of 420–500 °C. Furthermore, CNOs were produced at frequencies near the acoustically resonant frequency (60–70 Hz) for gas temperatures ranging between 620 and 720 °C. Chung and Lin [31] further examined the combined effects of acoustic excitation and nitrogen dilution on the synthesis of carbon nanomaterials in ethylene/air diffusion flames. The synthesis of CNOs was greatly affected by acoustic excitation at *f* = 10–20 Hz (near the natural flickering frequency) or at 60–70 Hz (near the acoustically resonant frequency) for ethylene concentrations equal to or greater than 60% at a height above the burner exit of *z* = 5 mm or equal to or greater than 40% at *z* = 10 mm.

CNTs and CNOs prefer different environments due to their own growth mechanisms. For the growth mechanism in flame synthesis in CNTs, it is well accepted that catalytic metallic nanoparticles are required [10]. The model was postulated by Baker [32] including (1) absorption of the hydrocarbons on the nanoparticle; (2) dehydrogenation of hydrocarbons and diffusion of carbon to form the solid layers on the particle; and (3) extrusion/precipitation of diffused carbon from the catalytic particle. In flame synthesis, the hydrocarbons are supplied from the decomposition of fuel. Flames under deficient oxygen conditions provide abundant hydrocarbons and mid–low temperature due to incomplete combustion. Light hydrocarbons (up to C2 species) and CO are considered possible species that contribute to the growth of CNT in flames [33]. The low temperature also prevents synthesized CNTs from being oxidized or burned. Thus, CNTs were more likely to synthesize in the weak and blue flames. In the experiments, CNOs were found to be strongly related to the presence of soot layers. The high concentrations of carbon vapor, C1, C2 and aromatic fragments inside soot layers can be considered as favorable building blocks of CNOs.

Most studies have focused on the flame synthesis of CNMs using a single fuel, and scant attention has been placed on the effect of mixed fuels on this synthesis [34]. In particular, less emphasis has been put on the fabrication of CNOs using the flame synthesis method. Evidently, there is still a need to explore this area because the morphologies, microstructures, and growth mechanisms of these materials are quite different from those for CNTs [35]. Moreover, the effect of flow mixing enhanced by acoustic excitation on CNO formation has not yet been well understood. Therefore, more effort needs to be devoted to studying and determining the proper synthesis conditions for CNO synthesis in mixed fuel diffusion flames modulated by acoustic excitation. In the present study, experiments are performed to examine the effects of the acoustic excitation frequency and mixed fuel (methane/ethylene ratio) on the fabrication and structure of carbon nanomaterials using laminar acoustically modulated jet diffusion flames.

## 2. Experimental Setup and Method

**Figure 1:**
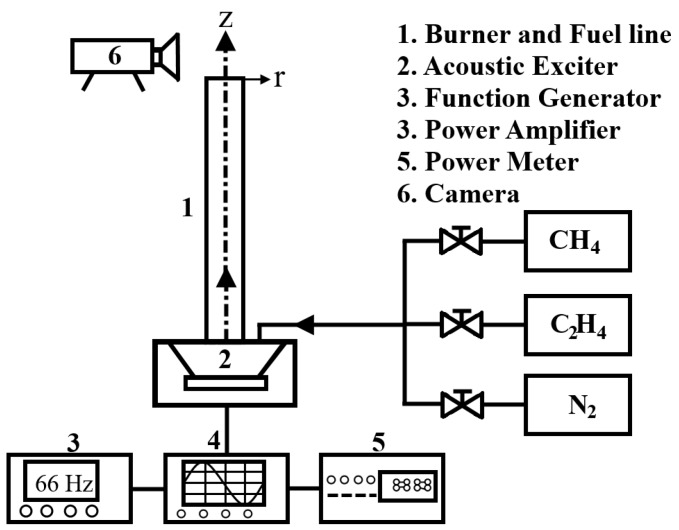
Schematic of acoustically modulated jet flow system.

The structure of synthesized carbon nanomaterials produced in mixed-fuel laminar jet diffusion flames with acoustic excitation was studied experimentally. This section describes the experimental setup (Figure 1) and method.

### 2.1. Acoustically Modulated Jet Flow Syetem

Figure 1 displays a schematic diagram of the acoustically modulated jet burner and associated apparatus used in this experiment. The experimental system consisted of a jet burner with a fuel line, an acoustic exciter, a function generator, a power amplifier, a power meter, and a camera (Nikon D70 digital camera, Nikon Corporation, Tokyo, Japan). The fuel, which consisted of methane and ethylene stored in high-pressure cylinders, was mixed in pre-specified volumetric concentrations (Ω_M_ and Ω_E_ both varied in the range of 0%–100%) by means of two flow meters. Typical flow rates used for both fuels were held at a constant velocity of 20 cm/s. The mixture of methane and ethylene was then allowed to flow and was excited by the acoustic modulator.

After mixing, the fuel flowed into an exciter, and the oscillation of the methane–ethylene flow was periodically modulated by an acoustic exciter incorporated into a forcing chamber composed of a hermetically sealed acrylic cube (24 × 24 × 13 cm^3^). The acoustic modulation system consisted of a function generator, power amplifier, power meter, and acoustic exciter. The acoustic drive in this system was performed by an 80 W loudspeaker with a diameter of 20.32 cm, which provided the desired excitation frequencies. The oscillation of the gas flow could be described by a sinusoidal signal, for which a function generator was used to produce signals with frequencies ranging from 0–90 Hz; afterwards, the signals were amplified by an amplifier. The powered signals were then used to drive the acoustic exciter. In our preliminary experiments, three acoustic powers of 5, 10 and 15 W were tested. At a lower acoustic power (5 W), the effect of acoustic excitation was weaker. At a higher acoustic power (15 W), much stronger suction and mixing in the flow field could be provided leading to the possible occurrence of flame extinction, especially for low-ethylene-percentage flames. Therefore, we chose the acoustic power of 10 W in this study. For each actuating signal, the output power was maintained at a constant value of 10 W using a power meter. Finally, the fuel flowed downstream, passed through the fuel line, and mixed with the ambient air.

The acoustically resonant frequency of the methane–ethylene flame can be calculated by Equation (1) [36,37]:

(1) $$f=n\frac{a}{2L},n=1,2,3\ldots ,$$

where a is the speed of sound in air (~323.3 m/s for ethylene and ~446 m/s for methane); *L* is the length of the fuel line (2.5 m), and *n* is the frequency mode (*n* = 1, in the present analysis). Hence, the first acoustically resonant frequency of ethylene was 66 Hz, while that of methane was 90 Hz.

The diffusion flame was supported on a jet burner consisting of a single stainless tube with a 1.1 cm inner diameter and a 1.3 cm outer diameter. The length of the burner tube was 45 cm, which helped in making a fully developed laminar velocity profile at the exit. As mentioned above, the burner had a 2.5-m-long fuel line. After passing through the acoustic exciter, the mixture of methane and ethylene used for generating the diffusion flame was introduced through a 1.1-cm-diameter fuel line and ignited at the burner exit.

### 2.2. Measurement and Sampling Systems

The flame structure was observed using a digital camera (Nikon D70). The temperature distribution along the axis of symmetry of the fuel line was measured using an R-type thermocouple (Pt/Pt-13% Rh and 0.05-inch diameter) driven by a 3D positioner. A nascent nickel mesh (200 mesh) with a diameter of 3 mm and a thickness of 0.2 mm was placed horizontally into the flame and served as the catalytic metal substrate to collect the deposited materials. In the synthesis experiments, the mesh was placed 10 mm above the burner exit plane along the axis (*r* = 0). The deposition time was specified as 120 s in every case. The focus of this study was on analyzing the formation of carbon nanomaterials sampled at *z* = 10 mm above the burner exit along the centerline for a fixed power output of 10 W. This is due to the fact that the flow field near the flame base (0 ≤ *z* ≤ 10 mm above the burner exit) was strongly affected by acoustic excitation. Hence, flame stability, the uniformity distribution of heat, and carbon precursors can be increased due to the partial premixing occurring at the flame base region [30]. As a consequence, enhanced synthesis of carbon nanomaterials could be observed. These facts caused the emphasis to be concentrated on the near flame base region in this study because the acoustic excitation effect was weakened in the downstream.

The deposited materials were characterized via field-emission scanning electron microscopy (FE-SEM, JEOL JSM-7000F, Tokyo, Japan) and high-resolution transmission electron microscopy (HR-TEM, JEOL JEM-2100). In addition, further observation was carried out to quantify the nano-materials produced, for which image tool software (Image J, version 1.45) was employed to analyze the diameter of the synthesized products.

## 3. Results and Discussion

### 3.1. Flame Structures

**Figure 2:**
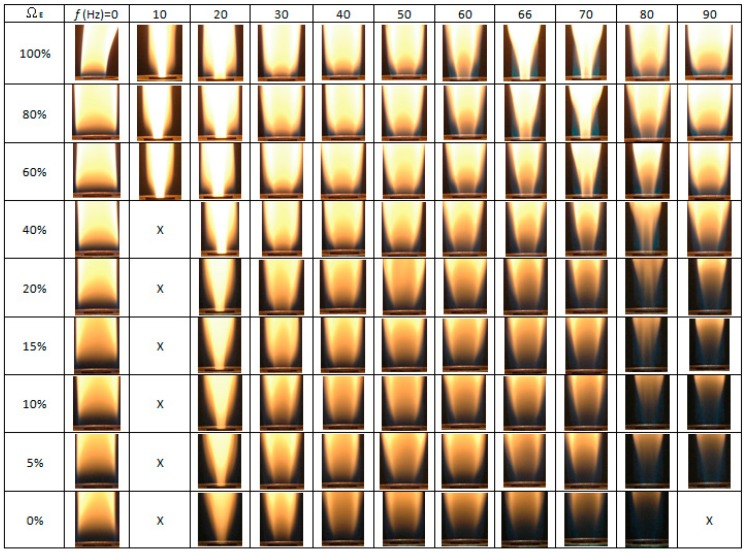
Effect of modulation frequency (*f*) and fuel concentration ratio (Ω_E_) on flame structure.

Figure 2 presents the experimental images of the flame structure obtained using excitation frequencies (*f*) in the range of 0–90 Hz and C_2_H_4_ concentrations (Ω_E_) in the CH_4_/C_2_H_4_ mixture ranging from 0% to 100%. As shown in Figure 2, for a frequency of *f* = 0 Hz, the flame has a single-layer structure for all values of Ω_E_. However, for frequencies in the range of 10–90 Hz, a double-layer flame structure can be observed, in which the outer flame has a bright appearance (wider than the burner exit) while the inner flame core is luminous (narrower than the burner exit).

For values of Ω_E_ less then 40% and a frequency of *f* = 10 Hz, the flame disappears (denoted by “X”). The flame also disappears at Ω_E_ = 0% (i.e., pure methane) and *f* = 90 Hz. In both cases, flame extinction occurs because the flame has a low intensity. In general, the low-frequency oscillation (10–15 Hz) or “flickering” of laminar diffusion flames is caused by buoyancy effects induced by Kelvin–Helmholtz instability [38].

The images presented in Figure 2 show that for modulation frequencies near both the flame flickering frequency (10–20 Hz) and acoustically resonant frequency (i.e., at *f* = 70 Hz for 60% ≤ Ω_E_ ≤ 100% and *f* = 80 Hz for 0% ≤ Ω_E_ ≤ 40%), the inner core was slimmer, and the flame was more luminous. Thus, the flame experienced acoustic excitation near the natural flickering frequency or acoustically resonant frequency could produce a bright core flame and the soot content could increase remarkably, compared with a steady flame without acoustic excitation. However, for all modulation frequencies other than the natural flickering frequency and acoustically resonant frequency, the inner flame diameter was increased, and the flame was less luminous. For instance, the acoustic resonant frequency for Ω_E_ = 100% (i.e., pure ethylene) occurs at *f* = 66 Hz. As shown in Figure 2, at Ω_E_ = 100% without acoustic excitation (*f* = 0 Hz), a single-flame structure was generated. As the excitation frequency increased from *f* = 0 Hz to the frequency close to the flame flickering frequency (10 and 20 Hz), a double-flame structure composed of a slender core flame and an outer yellow flame was observed. With an increase in excitation frequency from 10 to 40 Hz, the diameter of the core flame surface in the flame base region near the burner exit gradually increased and the flame color became less luminous since the frequency deviated from the natural flickering frequency progressively. However, when the excitation frequency was increased greater than 40 Hz and approached the resonant frequency, 66 Hz, the diameter of the core flame surface gradually decreased and the flame color became more luminous. At the acoustically resonant frequency, 66 Hz, a double-flame structure with the slenderest core flame and the broadest blue outer flame was observed. With further increasing excitation frequency (*f* = 70–90 Hz), the yellow core flame gradually moved outwards and approached the blue outer flame. The change in the acoustic frequency resulted in a significant difference in the flame structure, which, in turn, significantly influenced the growth of CNMs.

**Figure 3:**
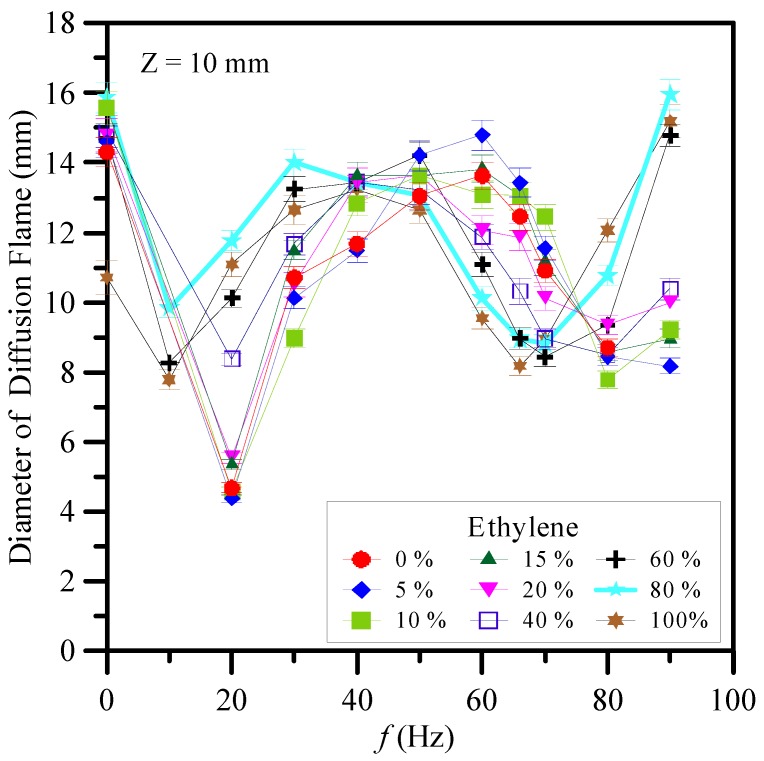
Variations of flame diameter at *z* = 10 mm with modulation frequency (*f*) and ethylene concentration (Ω_E_).

The heights of the diffusion flames were in the range of 21–25 cm. The observations in this paper were focused at *z* = 10 mm for all cases above the burner exit near the flame base region. The diameter of the diffusion flame is a significant parameter affecting the fabrication of CNMs at *z* = 10 mm. The slender and luminous core flame led to good synthesis of CNMs due to a favorable environment temperature and a suitable carbon precursor concentration. Variations of flame diameter at *z* = 10 mm with modulation frequency (*f*) and ethylene concentration (Ω_E_) are shown in Figure 3. As can be seen, the slenderest and most luminous core flame corresponding to the smallest diameter occurred at acoustic excitation near the natural flickering frequency or acoustically resonant frequency. It is noteworthy that the double-flame structure close to the burner exit was generated due to the reverse flow caused by the acoustic excitation. The reverse flow induced strong air entrainment, which slenderized the core yellow flame. Similar trends can be found for other fuel ratios.

The present experiments were performed using a CH_4_/C_2_H_4_ diffusion flame, and thus the acoustically resonant frequency was different from that of a single fuel only. For the pure ethylene flame (Ω_E_ = 100%), the resonant frequency occurred at 66 Hz, while for the pure methane flame (Ω_E_ = 0%), the resonant frequency occurred at 90 Hz, which coincide with the theoretical prediction of Equtation (1) [36,37]. As shown in Figure 2, the flame type at the frequencies 66 ≤ *f* ≤ 70 Hz was similar to that obtained for a modulation frequency in the range of 10 ≤ *f* ≤ 20 Hz and an ethylene concentration Ω_E_ ranging from 60% to 100%. Under these excitation and fuel concentration conditions, a significant amount of air was sucked into the burner exit during the entrainment part of the cycle. As a result, the jet flow was compressed, and the core flame burned more brightly [39].

There are two modes of resonance associated with acoustic excitation, i.e., natural flickering and acoustical resonance [31]. Natural flickering occurs for accoustic excitation frequencies in the range of 10–20 Hz due to the interaction between the hot combustion gas and the ambient air, which induces large vortices. Meanwhile, in acoustical resonance, a large amount of ambient air is entrained into the burner exit and mixes with the fuel. The acoustically resonant frequencies of the various CH_4_/C_2_H_4_ mixtures considered in this study are different since the sound speeds of ethylene and methane are different. As described above, based on the theoretical prediction, the resonant frequency is around 66 Hz for ethylene and 90 Hz for methane. The change in the acoustically resonant frequency leads to a significant difference in the flame structure and appearance.

The flame structures shown in Figure 2 can be classified into three main types: natural flickering, non-resonance, and acoustical resonance. As the ethylene concentration decreased, the blue part of the flame base thickened and the inner core became less luminous. At the acoustically resonant frequency, a double-layer flame structure was observed. Both the natural flickering mode and the acoustical resonance mode resulted in a flame with an inner luminous core, which was carbon-rich and had a high temperature. Furthermore, it was expected that the most suitable fuel concentration range for synthesis was wider at these two frequencies than at other frequencies [31]. Since the acoustically resonant frequency for different values of Ω_E_ was changed, the suitable range for CNM synthesis was smaller than that of natural flickering frequency. In general, natural flickering frequency caused by buoyancy effects provided a wider range for the CNM formation. Thus, it may be said that natural flickering frequency produces more favorable conditions for the synthesis of carbon nanostructures.

### 3.2. Temperature Measurements

**Figure 4:**
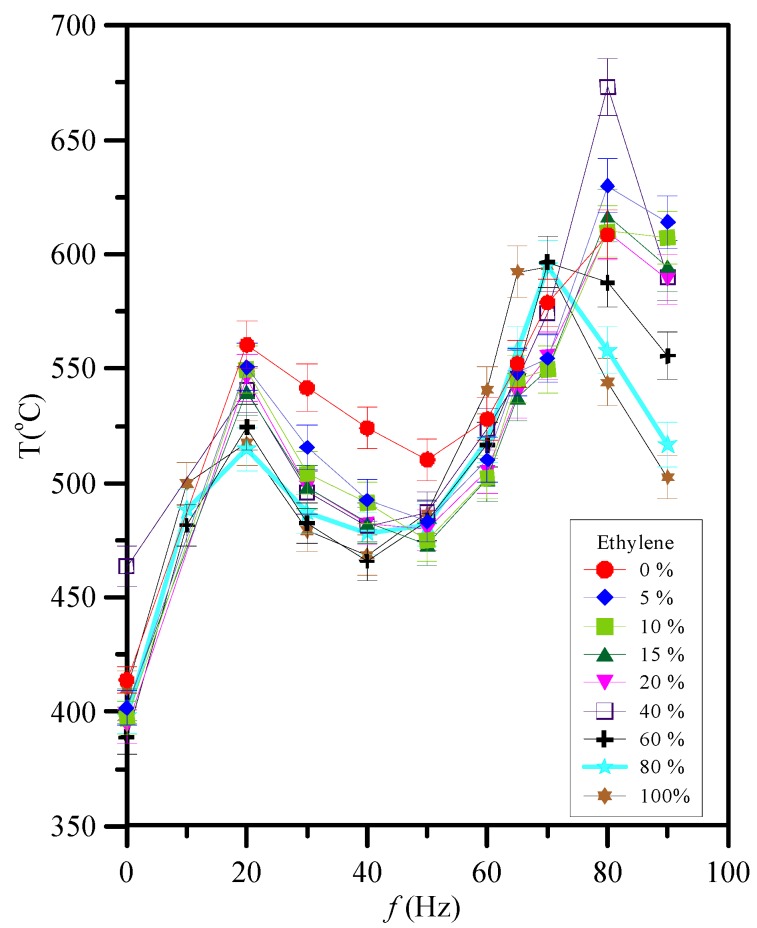
Mean temperatures (*T*) at *z* = 10 mm for various modulation frequencies (*f*) and ethylene concentrations (Ω_E_).

One of the important factors in nanostructure synthesis is the temperature of the environment. This investigation focused on the near-flame-base of the flame; hence, the mean temperatures (*T*) at *z* = 10 mm above the burner exit for an acoustic excitation 0–90 Hz and 0% ≤ Ω_E_ ≤ 100% were examined. The results are presented in Figure 4.

According to the measurement results displayed in Figure 4, two temperature peak values appeared at frequencies either as natural flickering or as an acoustically resonant frequency as the excitation frequency increased. With increasing Ω_E_, the highest temperature value decreased. For all values of ethylene concentration Ω_E_, the first peak always appeared at *f* = 20 Hz. The value of the temperature peak was around 510–560 °C at *f* = 20 Hz. However, the acoustically resonant frequency for different values of Ω_E_ was changed. For 0% ≤ Ω_E_ ≤ 40%, the acoustically resonant frequency occurred at about 80 Hz, while, for 60% ≤ Ω_E_ ≤ 100%, it was close to 70 Hz. The second peak value appearing at acoustically resonant frequency (*f* = 80 Hz) was about 600–675 °C for 0% ≤ Ω_E_ ≤ 40%, while it was approximately 600 °C for 60% ≤ Ω_E_ ≤ 100% (at *f* = 70 Hz). Figure 4 also shows that, for a fixed Ω_E_ without acoustic excitation (*f* = 0 Hz), the gas temperature at the sampling position (*z* = 10 mm) was low because of the broad single flame structure. On the contrary, at *z* = 10 mm, the gas temperature of the flame with acoustic excitation was higher than that without acoustic excitation due to a slenderer core flame. The highest temperature would appear at the acoustically resonant frequency. The resonant frequency for 40% ethylene is very close to 80 Hz such that the highest temperature occurred at 80 Hz because of the slenderest core flame caused by the acoustic excitation. Moreover, it can be inferred that the suitable frequencies for synthesis of carbon nanostructures were around *f* = 20 Hz for 0% ≤ Ω_E_ ≤ 100%; *f* = 80 Hz for 0% ≤ Ω_E_ ≤ 40%; and *f* = 70 Hz for 60% ≤ Ω_E_ ≤ 100%.

### 3.3. Flame Synthesis of Carbon Nanomaterials

The focus of this study was on analyzing the formation of carbon nanomaterials sampled at *z* = 10 mm above the burner exit along the centerline for a fixed power output 10 W. This is due to the fact that the flow field near the flame base (0 ≤ *z* ≤ 10 mm above the burner exit) was strongly affected by acoustic excitation. The natural convection from buoyancy is stronger than the forced convection in the downstream region, so the tip of the flame usually vibrates [31]. Moreover, rich carbon particles accumulate on a thin flame front. These situations indicate that the near flame front region may not provide suitable conditions for stable nanomaterial synthesis. Accordingly, a diffusion flame was employed to enhance the air entrainment to allow good flow mixing by means of acoustic excitation. As a consequence, flame stability, the uniformity distribution of heat, and precursors can be increased due to the partial premixing occurring at the flame base region. These facts cause the emphasis to be concentrated on the near flame base region in this study because the acoustic excitation effect was weakened in the downstream. The mean temperatures were higher at *z* = 10 mm; hence, high-density carbon nanomaterials were formed [31]. Therefore, the observations in this paper were focused on *z* = 10 mm for all cases.

**Figure 5:**
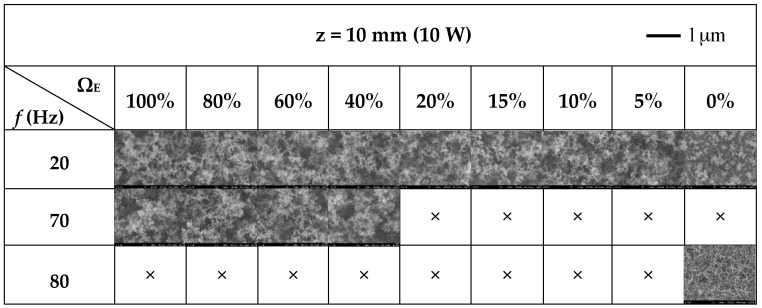
Scanning electron microscopy (SEM) images of carbon nanomaterials synthesized at *z* = 10 mm for various values of Ω_E_ and *f*. ×: No CNMs; CNMs: carbon nanomaterials.

In the experiments, scanning electron microscopy (SEM), which is able to produce images by using electrons instead of light, was employed to analyze the formation of the synthesized carbon nanomaterials. A sample can be magnified at high levels with SEM. Therefore, the quantity of carbon nanostructures can be displayed. Due to the change in electric concentration at the tip of nanostructures occurring in a high-voltage SEM environment, the substrate surface is a deep gray color, and the nanoparticles are marked in white color [31]. The formation of carbon nanomaterials was greatly affected by acoustic excitation at frequencies near the natural flickering frequency and the acoustically resonant frequency. Based on the flame structure and the temperature measurement shown in Section 3.1 and Section 3.2, the results for nanoparticles sampled at *z* = 10 mm and 0% ≤ Ω_E_ ≤ 100% for frequencies 20, 70 and 80 Hz are presented in Figure 5.

**Figure 6:**
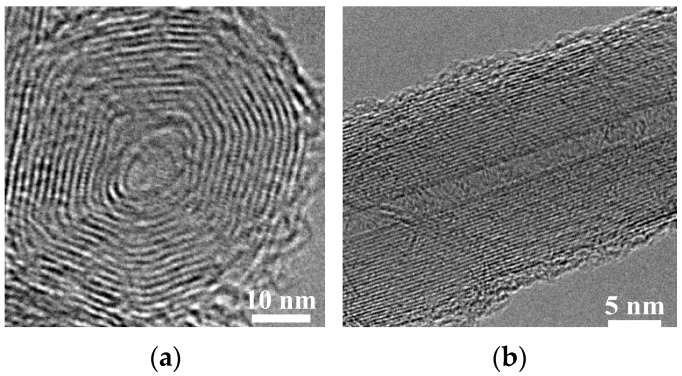
Typical transmission electron microscopy (TEM) images of carbon nanomaterials synthesized at *z* = 10 mm: (**a**) carbon nano-onion (CNO), and (**b**) carbon nanotube (CNT).

As shown in Figure 5, there was a large quantity of carbon nanomaterials (piled like bunches of grapes) on the substrate at near natural flickering frequency *f* = 20 Hz for all values of fuel ratio (vol % ethylene Ω_E_). For frequencies near the acoustically resonant frequency *f* = 70 Hz, a large quantity of carbon nanomaterials was formed at 40% ≤ Ω_E_ ≤ 100%. Under these synthesis conditions, the carbon nanomaterials piled up like bunches of grapes. The SEM images show that this microstructure is sphere-like, and the TEM image in Figure 6a shows that the synthesized product is the so-called carbon nano-onion (CNO). However, the products decreased abruptly at 0% ≤ Ω_E_ ≤ 20%. From these results, it was verified that the main products are CNOs and that the flame structure significantly affects the formation of carbon nanomaterials. The slender, luminous inner flame core led to good synthesis of carbon nanostructures due to the favorable environmental temperature and high carbon precursor concentration.

When the excitation frequency was increased to 80 Hz, no carbon nanomaterial was produced at 5% ≤ Ω_E_ ≤ 100% for 10 W. However, a large quantity of CNTs was fabricated at *f* = 80 Hz for Ω_E_ = 0% (pure methane). A typical TEM image of CNT under these conditions is shown in Figure 6b. The acoustically resonant frequency for methane is 90 Hz. At *f* = 80 Hz (close to the acoustic resonant frequency), both the temperature and the concentration of carbon precursors were appropriate and favorable for CNT growth and thus provided a suitable environment for the synthesis of CNTs.

Table 1 shows the production yield of CNMs under different excitation frequencies and ethylene concentrations. Darker shades indicate higher yields. Based on SEM observations (Figure 5) and Table 1, we can compare the production yield of CNMs under different experimental conditions. It is found that high-yield synthesis of CNOs could be achieved at *f* = 20 Hz for 5% ≤ Ω_E_ ≤ 100% and at *f* = 70 Hz for 40% ≤ Ω_E_ ≤ 100%. In addition, moderate-yield synthesis of CNOs could be observed at *f* = 20 Hz and Ω_E_ = 0%. Moreover, a pure methane flame (Ω_E_ = 0%) experiencing acoustic excitation (*f* = 80 Hz) close to acoustically resonant frequency could yield high density of CNTs.

It is noticed that, in the present study, a large quantity of CNOs was formed at *f* = 20 Hz for 0% ≤ Ω_E_ ≤ 100%, with a temperature range of 510–560 °C and at *f* = 70 Hz for 40% ≤ Ω_E_ ≤ 100%, with a temperature range of 575–600 °C. In these experimental conditions, the flame structure was composed of a slender yellow core flame. On the other hand, CNTs were fabricated only at *f* = 80 Hz for Ω_E_ = 0% (very close to the acoustically resonant frequency for methane) in a blue core flame with a gas temperature of 600 °C. It is interesting to note that CNOs were synthesized in a sooty yellow core flame, whereas CNTs were fabricated in a blue core flame. The slender and luminous core flame led to good synthesis of CNMs due to a favorable environment temperature and a suitable carbon precursor concentration.

At *f* = 80 Hz, when Ω_E_ is in the range of 10%–40%, the core flames were sooty with yellow color but less sooty than those at *f* = 20 Hz for 0% ≤ Ω_E_ ≤ 100% and *f* = 70 Hz for 40% ≤ Ω_E_ ≤ 100%. Moreover, in these experimental conditions, the temperature was higher than the temperature range suitable for the synthesis of CNOs. As a result, CNOs could not be successfully synthesized at *f* = 80 Hz for 10% ≤ Ω_E_ ≤ 100%. At *f* = 80 Hz and Ω_E_ = 5%, the core flame was blue. However, it provided too many carbon precursors and/or too high temperatures that were not suitable for the fabrication of CNTs, compared with that of *f* = 80 Hz and Ω_E_ = 0%. Consequently, CNTs were not observed at *f* = 80 Hz and Ω_E_ = 5%. In this study, at *f* = 80 Hz (close to the acoustic resonant frequency) and Ω_E_ = 0% (pure methane), the temperature and the concentration of carbon precursors at *z* = 10 mm provided a suitable environment for the synthesis of CNTs. That is, both the temperature and carbon precursor concentration at *z* = 10 mm were appropriate and favorable for CNT formation. It is noteworthy that CNOs were synthesized in a sooty yellow core flame, whereas CNTs were fabricated in a blue core flame. The slender and luminous core flame led to good synthesis of carbon nanomaterials (CNMs) due to a favorable environment temperature and a suitable carbon precursor concentration. Therefore, both heat source (temperature) and carbon source (carbon precursor concentration) dominate the synthesis of CNMs.

**Figure 7:**
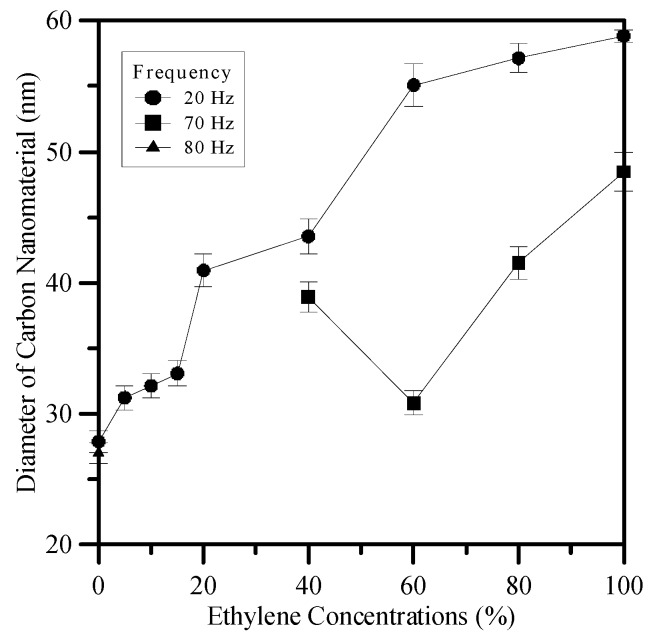
Variations of average diameters of synthesized carbon nanomaterials with Ω_E_ for *f* = 20, 70 and 80 Hz at *P* = 10 W.

The average diameters of the carbon nanomaterial are shown in Figure 7. In general, the average diameter of CNOs formed at 20 Hz was slightly greater than that produced at 70 Hz due to the intense mixing at higher frequency that resulted in a smaller diameter. The average diameter of CNOs fabricated in counterflow diffusion flames is dependent on the concentration of methane [17]. In this study, the average diameters of the CNOs synthesized in jet diffusion flames increased with decreasing methane concentrations. The diameter tended to decrease with decreases in Ω_E_. The average diameters at 70 Hz corresponding to ethylene concentrations of 40%, 60%, 80%, and 100% were approximately 39, 30, 42, and 48 nm, respectively. At the natural flickering frequency of 20 Hz, the average diameters decreased gradually from 59 nm at Ω_E_ = 100% to 28 nm at Ω_E_ = 0%, as shown in Figure 7. The CNTs produced at 80 Hz for Ω_E_ = 0% have an average diameter of 27 nm and lengths up to 1.2 μm.

## 4. Conclusions

This study was conducted to examine the effects of acoustic modulation and mixed fuel (blends of methane–ethylene) on the synthesis of carbon nanomaterials in a laminar jet diffusion flame. The results showed that a single-layer flame structure was produced without acoustic excitation (*f* = 0 Hz) for all values of Ω_E_, while a double-layer flame structure was generated for frequencies near both the natural flickering frequency and the acoustically resonant frequency. Moreover, the flame disappeared for values of Ω_E_ less than 40% at *f* = 10 Hz and for Ω_E_ = 0% at *f* = 90 Hz.

The synthesis of carbon nano-onions was significantly enhanced by acoustic excitation near the natural flickering frequency and the acoustically resonant frequency. Under these two excitation conditions, a double-layer flame structure was formed with a slender inner core and a more luminous (i.e., higher temperature) flame. This double-layer flame structure provided a favorable flame environment for the fabrication of carbon nanomaterials. It was noticed that a large quantity of CNOs was formed at *f* = 20 Hz for 0% ≤ Ω_E_ ≤ 100%, with a temperature range of 510–560 °C and at *f* = 70 Hz for 40% ≤ Ω_E_ ≤ 100%, with a temperature range of 575–600 °C. Carbon nanotubes were fabricated only at *f* = 80 Hz for Ω_E_ = 0% (close to the acoustically resonant frequency for methane) with a gas temperature of 600 °C. However, with the exception of these cases, almost no carbon nanomaterials were formed.

## Figures and Tables

**Table 1:** Production yield of the carbon nanomaterials under different experimental conditions.

	Ω_E_	100%	80%	60%	40%	20%	15%	10%	5%	0%
*f* (Hz)										
20		CNOs	CNOs	CNOs	CNOs	CNOs	CNOs	CNOs	CNOs	CNOs
70		CNOs	CNOs	CNOs	CNOs	×	×	×	×	×
80		×	×	×	×	×	×	×	×	CNTs

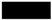
: High-yield CNMs; 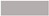: Moderate-yield CNMs; ×: No CNMs. CNOs: Carbon nano-onions; CNTs: Carbon nanotubes; CNMs: Carbon nanomaterials.
